# Evolutionary dynamics of rhomboid proteases in *Streptomycetes*

**DOI:** 10.1186/s13104-015-1205-x

**Published:** 2015-06-09

**Authors:** Peter A Novick, Naydu M Carmona, Monica Trujillo

**Affiliations:** Biological Sciences and Geology Department, Queensborough Community College, City University of New York, Bayside, NY USA

**Keywords:** *Streptomyces*, Rhomboid proteins, Proteases, *S. coelicolor*, Bioinformatics

## Abstract

**Background:**

Proteolytic enzymes are ubiquitous and active in a myriad of biochemical pathways. One type, the rhomboids are intramembrane serine proteases that release their products extracellularly. These proteases are present in all forms of life and their function is not fully understood, although some evidence suggests they participate in cell signaling. *Streptomycetes* are prolific soil bacteria with diverse physiological and metabolic properties that respond to signals from other cells and from the environment. In the present study, we investigate the evolutionary dynamics of rhomboids in *Streptomycetes,* as this can shed light into the possible involvement of rhomboids in the complex lifestyles of these bacteria.

**Results:**

Analysis of *Streptomyces* genomes revealed that they harbor up to five divergent putative rhomboid genes (arbitrarily labeled families A–E), two of which are orthologous to rhomboids previously described in *Mycobacteria*. Characterization of each of these rhomboid families reveals that each group is distinctive, and has its own evolutionary history. Two of the *Streptomyces* rhomboid families are highly conserved across all analyzed genomes suggesting they are essential. At least one family has been horizontally transferred, while others have been lost in several genomes. Additionally, the transcription of the four rhomboid genes identified in *Streptomyces coelicolor*, the model organism of this genus, was verified by reverse transcription.

**Conclusions:**

Using phylogenetic and genomic analysis, this study demonstrates the existence of five distinct families of rhomboid genes in *Streptomycetes*. Families A and D are present in all nine species analyzed indicating a potentially important role for these genes. The four rhomboids present in *S. coelicolor* are transcribed suggesting they could participate in cellular metabolism. Future studies are needed to provide insight into the involvement of rhomboids in *Streptomyces* physiology. We are currently constructing knock out (KO) mutants for each of the rhomboid genes from *S. coelicolor* and will compare the phenotypes of the KOs to the wild type strain.

## Background

Rhomboids are intramembrane serine proteases, mechanistically characterized because they release factors to the outside of the cell, rather than into the cytosol. The first rhomboid protease was discovered in *Drosophila* [1] and further work revealed the existence of homologs in all branches of life [2]. The human and mouse genomes encode rhomboid genes, but the largest number of rhomboid genes are encoded by plants [2]. Despite their ubiquity, rhomboid sequence identity is only about 6% across all domains of life [2]. This extremely low conservation could be due to the fact that sequences are predominantly transmembrane, thereby experiencing a different evolutionary pressure than other proteases [2]. Through multidisciplinary approaches, it has been demonstrated that rhomboids create a central hydrated microenvironment immersed below the membrane surface; this microenvironment supports the hydrolysis carried out by its serine protease-like catalytic apparatus [3]. Rhomboid proteases can have three different topologies. The simplest form has six transmembrane (TM) helices and it is the most prevalent in prokaryotic rhomboids. The other two forms have an extra TM helix either at the C-terminus (6 + 1 TM) or at the N-terminus (1 + 6 TM), and are typical of eukaryotic rhomboids. Prokaryotic rhomboids AarA from the pathogenic *Providencia stuartii*, YqgP from *Bacillus subtilis* and Rv0110, one of the rhomboids from *Mycobacteria* are exceptions as they exhibit 6 + 1 TM topology like eukaryotes [4].

In spite of their shared mechanism of action, rhomboids exhibit a wide diversity of biological roles. It has been demonstrated that these proteases: control EGF receptor signaling in *Drosophila* and *Caenorhabditis elegans* [2], are involved in the cleavage of adhesins in apicomplexan parasites, and regulate aspects of mitochondrial morphology and function in yeast and multicellular eukaryotes [5]. Furthermore, rhomboids are involved in processes such as inflammation and cancer suggesting they could have therapeutic potential [6]. Not much is known to date about the biological role that rhomboids play in prokaryotes. AarA, from *P. stuartii* affects quorum sensing [7], YqgP from *B. subtilis* plays a role in cell division and glucose uptake [8]. The crystal structure of GlpG from *Escherichia coli* has been solved, yet its biological function is still undetermined [9]. Rhomboid proteases, Rv0110 and Rv1337 from *Mycobacterium*, a genus from the Actinomyces phylum, have been described, yet their substrates have not been identified [10, 11]. Little is also known about rhomboids from *Streptomyces*, another genus from Actinomyces [11]. *Streptomycetes* are “multicellular” prokaryotic organisms with a complex developmental cycle and secondary metabolite secretion, in which signals from other cells and from the environment are detected, integrated and responded to using multiple signal transduction systems.

*Streptomycetes* produce two thirds of all industrially manufactured antibiotics, and a large number of eukaryotic cell differentiation-inducers [12], apoptosis inhibitors [13] and inducers [14–16], protein C kinase inhibitors [17], and compounds with antitumor activity [18]. The production of these secondary metabolites (physiological differentiation) is usually temporally and genetically coordinated with the developmental program (morphological differentiation) when cultured in agar and is likely to respond to environmental, physiological and stress signals [19–22]. These processes are controlled by different families of regulatory proteins, and are elicited by both extracellular and intracellular signaling molecules mediated by an array of signal transduction systems [23]. Given the involvement of rhomboids in cell signaling, we propose that they could participate in some of the signaling cascades existing in *Streptomycetes*.

Here, we use bioinformatics to identify and describe putative rhomboid genes in the genome of nine fully-sequenced *Streptomyces* species [24–32]. Furthermore, we demonstrate that these genes are transcribed in *Streptomyces coelicolor*, the model organism for this genus.

## Methods

### Sequence analysis

Nucleotide and amino acid sequences from *Streptomycetes* and related species were collected in two ways to guarantee an exhaustive search. An initial collection was obtained from a BLASTp search using a previously identified rhomboid protein from *P. stuartii*, aAaR (S70_10405) as the query. Secondly, a gene search using the Integrated Microbial Genomes Education Site (IMG/EDU) was conducted retrieving all genes with the pfam01694 domain [33]. Using a combination of both NCBI and IMG, we determined which genomes have complete sequence data, and which are in draft format. We limited our final analysis to nine completely sequenced genomes. All resulting sequences were aligned in Bioedit using ClustalW and sorted based upon sequence similarity [34]. An initial neighbor joining phylogenetic tree was constructed using the translated nucleotide sequences which aided in the construction of our groups. Monophyletic families with high similarity and bootstrap support (>80%) were then arbitrarily named A–E.

Active sites in the rhomboid domain were found using pfam and e values were collected for support [35]. Any additional domains identified were collected. Using TMHMM and Phobius, sequences were analyzed for the number of transmembrane domains, amount of transmembrane amino acids, the cellular locations of active sites and the distance between them [36, 37]. A two-dimensional model of the transmembrane structure was constructed using TMRPres2D [38].

In order to determine conserved sites within and across families, sequences were analyzed with weblogo which indicates with large letters residues that are most likely important in protein function [39]. Consensus sequences of each family were also constructed using Bioedit and the pair wise divergence of the rhomboid domains within and between each family was calculated to determine which families were most similar.

### Phylogeny reconstruction

One sequence from each family was used as a query for a BLASTp search of all sequenced genomes excluding the Actinomycetes [40]. Then, a phylogenetic tree containing sequences from our library and the non-Actinomycetes sequences were constructed to determine other species that harbor the same family in their genome.

Mega 5.0 was utilized for our phylogenetic reconstructions [41]. Previously aligned sequences were used to construct both neighbor joining trees and maximum likelihood trees for comparison. 1,000 bootstrap replicates were calculated and those lower than 70 were removed.

### Gene neighborhoods

IMG-ACT was used to determine the genome location of each rhomboid gene. Using the five sequences from *S. scabiei* as a query, all orthologs were identified to determine if they were found in a similar region of the genome, in the same orientation, and had the same neighboring genes. The presence or absence of operons and the functions of neighboring genes were also determined [33].

### Strains and cultures

*S. coelicolor* M145 was kindly provided by Dr. Mervyn Bibb. *S. coelicolor* was grown in Mannitol Soya flour and in R5 medium [42].

### PCR conditions

Chromosomal DNA was extracted from *S. coelicolor* [42]. Internal primers for the four putative rhomboid genes for *S. coelicolor* were designed with Primer 3 [43] (Table 1). Amplification reactions contained 0.3 μM each of the rhomboid specific forward and reverse primers (Table 1), 0.02 U/μl KOD Hot Start Master Mix (Novagen) ~200 ng genomic DNA and nuclease free water in a reaction volume of 20 μl. The reactions were performed in a C1000 Thermocycler (BioRad) using the following conditions: Initial polymerase activation and denaturation was done at 95°C for 2 min, followed by 30 cycles consisting of: denaturation at 95°C for 20 s, annealing at 60°C for 10 s, extension at 70°C for 10 s with a final extension at 70°C of 5 min. The correct internal fragment size was amplified for the four putative rhomboid genes.

**Table 1:** Primer sequences

Gene identifier	Primer sequence	Fragment size (bp)
SCO3855 family A	GCTACCTCGCCCTCTACCTC GGGTGAAGGTGAAGATCAGG	206
SCO2013 family B	GATGAACATGGTCGTGCTGT GGCCATGAAACGGTTGAC	241
SCO6038 family C	GCTCTTCCTGTGGATCTTCG CCTCGGATACAGCACCAGAT	196
SCO2139 family D	CTGCCCTTCCTCTTCTTCCT ACGCCACCAACTCTAGTCGT	197

### Transcription assays

50 ml of R5 [44] liquid media in a 250 ml flask were inoculated with spores from a Soy Flour Manitol [44] *S. coelicolor* plate and incubated in 30°C shaker at 240 rpm for 2 days. The pellet was harvested and RNA was prepared using EZRNA Total RNA Kit. Reverse transcription was done using M_MLB Reverse Transcriptase, oligo-random primers and nucleotide mixture from Promega, with ~60 ng/μl mRNA used as template. PCR was performed using the validated primers (Table 1) following the protocol described above. The expected fragment sizes were revealed via gel electrophoresis, the purified PCR product was cloned into pUC19, and DNA sequences were verified by sequencing at DNA analysis facility (Yale University).

## Results and discussion

### A. Phylogenetic studies and family relationships

**Figure 1:**
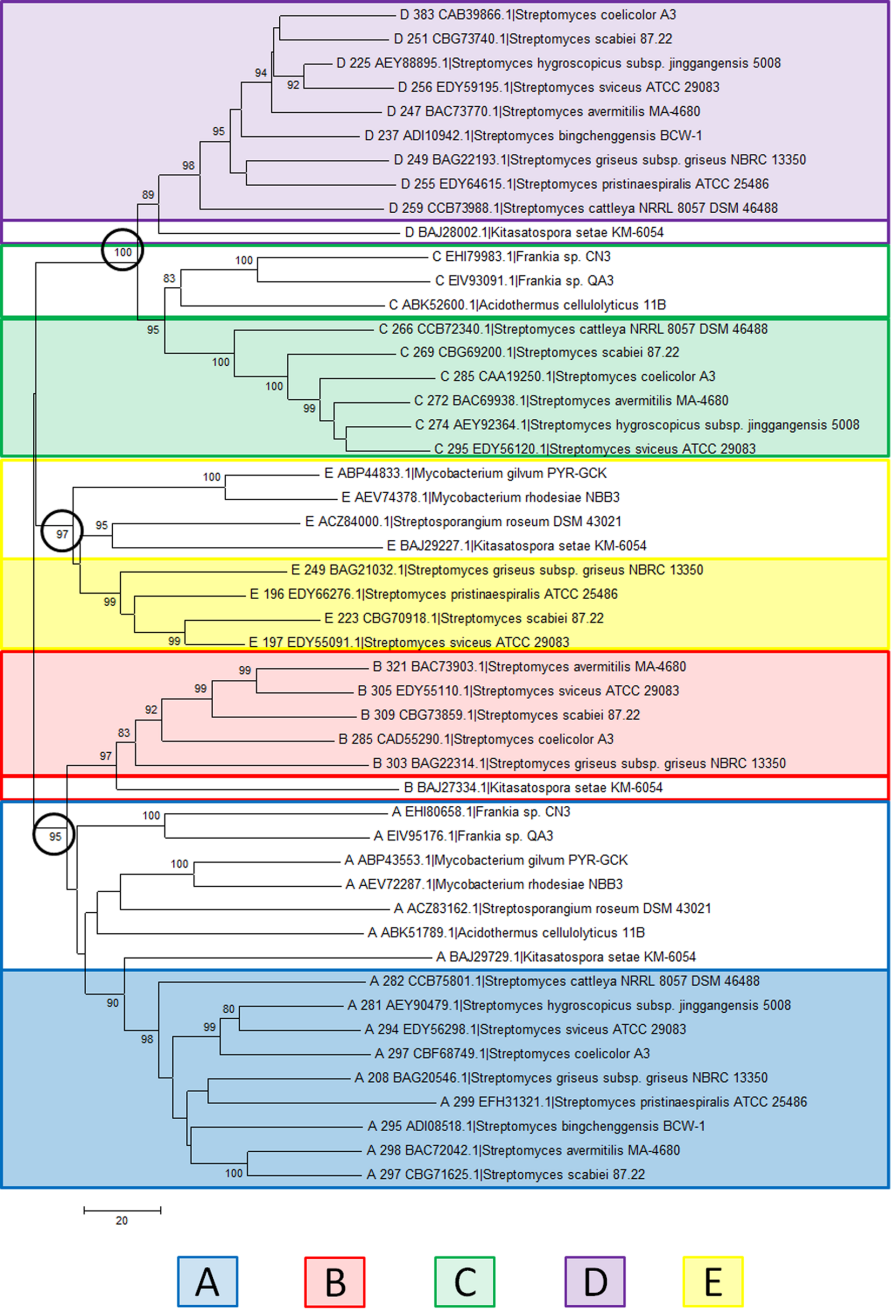
NJ Phylogenetic tree of the five putative rhomboid families using Mega 5.0 (Bootstrap values <70 have been omitted). *Colored boxes* are indicative of rhomboid sequences from *Streptomycetes*, while the *clear boxes* are sequences of the same rhomboid family, but from other *Actinomycetes*.

**Figure 2:**
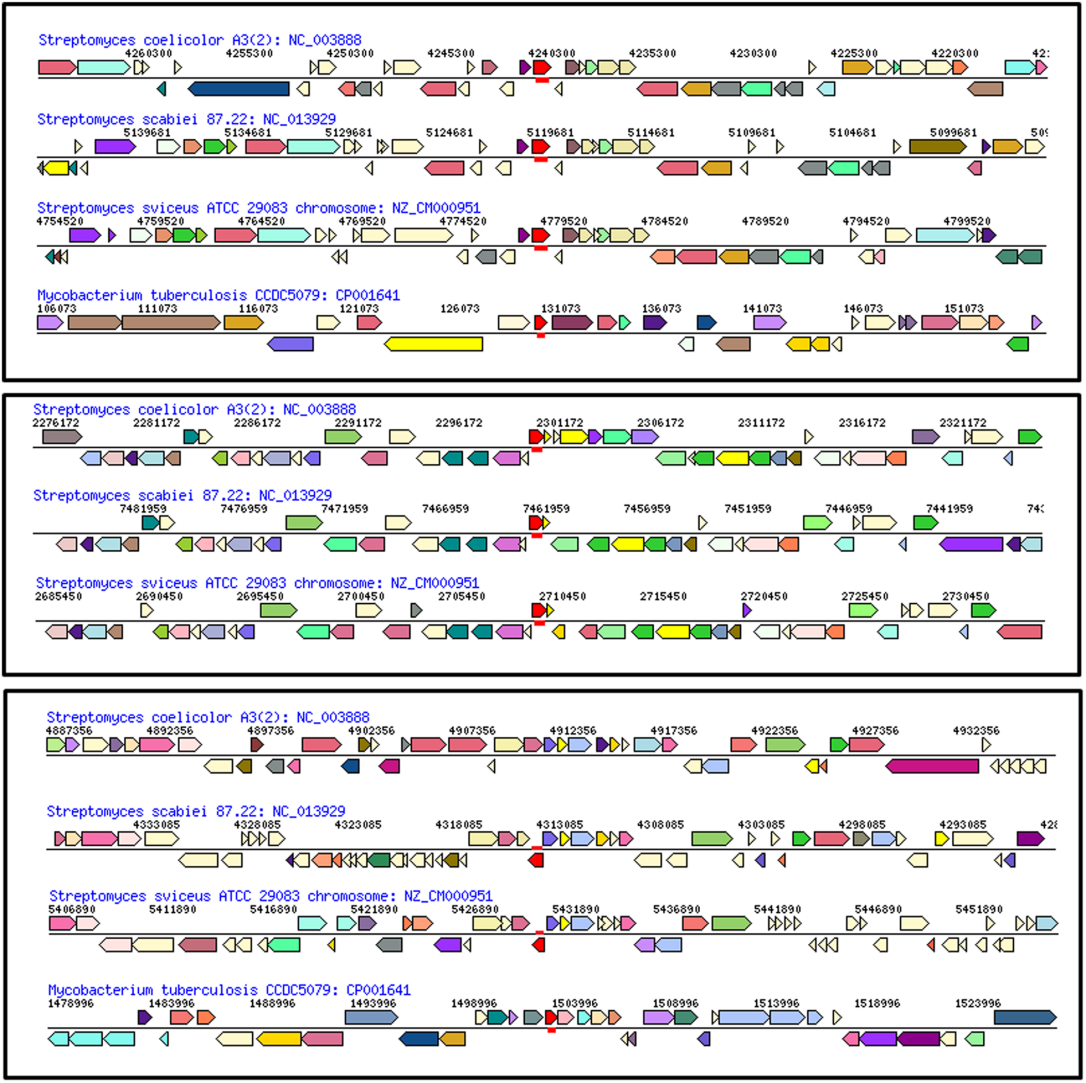
Gene neighborhoods for selected *Streptomyces strains* and *M. tuberculosis*. From *top* Family A, D and E. Rhomboid genes are *colored* in *red* and *underlined* when present.

The in silico analysis of rhomboid proteases in nine fully sequenced and assembled genomes of *Streptomycetes* revealed that their genomes have up to five diverse and distinct rhomboid genes (arbitrarily labeled Families A–E) as shown in Table 2. This is supported by high bootstrap values (Figure 1) and the high divergence across the five rhomboid families (Table 3). *Streptomyces avermitilis*, *bingchenggensis*, *cattleya*, *coelicolor*, *griseus*, *hygroscopicus*, *pristinaespiralis*, *scabiei* and *sviceus* have two to five rhomboid genes. Only *S. scabiei* and *S. sviceus* (Table 2) genomes contained all five families. Two of these families (A and D) are consistently present in the strains listed above, as well as in 30 additional *Streptomyces* genomes recently analyzed (data not shown). *Streptomyces* rhomboids have a few differences with the recently described *Mycobacterium* (a closely related Actinomycetes genus) rhomboids: *Streptomyce*tes harbor up to five rhomboids genes whereas *Mycobacteria* have a maximum of two; *Streptomyces* rhomboids are not found in large operons as the *Mycobacterium* counterparts are [10], and *Streptomyces* rhomboid genes also appear in different gene neighborhoods than *Mycobacterium* rhomboids (Figure 2). A summary of the phylogenetic analysis of the five rhomboid genes is presented (Figure 1).

**Table 2:** Analysis of putative rhomboid proteases from nine sequenced *Streptomycetes*

IMG Gene_OID	Species	Length (aa)^a^	Family	AS^b^	AS distance (aa)	aa in TMH^c^	# TMH	pfam e value
29607986	*S. avermitilis*	298	A	249, 203	46	130.58	6	2.50E−34
297158806	*S. bingchenggensis*	295	A	245, 199	46	132.73	6	2.10E−35
337767090	*S. cattleya*	282	A	232, 185	47	141.11	7	1.20E−34
**259419993**	***S. coelicolor*** **(** ***SCO3855*** **)**	**297**	**A**	**248, 202**	**46**	**138.3**	**7**	**3.30E−37**
178466026	*S. griseus* subsp*. griseus*	208	A	236, 190	46	136.70	7	4.50E−35
374101595	*S. hygroscopicus jinggangensis*	281	A	232, 186	46	121.77	6	2.60E−36
297193265	*S. pristinaespiralis*	299	A	249, 203	46	136.04	7	2.90E−37
260648514	*S. scabiei*	297	A	248, 201	47	135.09	7	1.70E−38
297200957	*S. sviceus*	294	A	245, 199	46	130.14	7	8.60E−36
29609854	*S. avermitilis*	321	B	270, 223	47	135.72	7	3.80E−27
**24419018**	***S. coelicolor*** **(** ***SCO2023*** **)**	**285**	**B**	**229, 182**	**47**	**139.55**	**7**	**9.70E−24**
178467794	*S. griseus* subsp*. griseus*	303	B	251, 204	47	131.42	6	1.20E–28
260650743	*S. scabiei*	309	B	258, 211	47	133.61	7	8.40E–26
197711076	*S. sviceus*	305	B	252, 205	47	138.69	7	1.10E–26
29605874	*S. avermitilis*	272	C	235, 169	66	128.22	6	1.70E–36
337763630	*S. cattleya*	266	C	235, 169	66	126.73	6	6.10E–35
**21224370**	***S. coelicolor*** **(** ***SCO6038*** **)**	**285**	**C**	**346, 280**	**66**	**132.24**	**6**	**2.60E−36**
374103480	*S. hygroscopicus jinggangensis*	274	C	235, 169	66	129.53	6	6.40E–37
260646107	*S. scabiei*	269	C	232, 166	66	128.35	6	2.50E–38
297203088	*S. sviceus*	295	C	256, 190	66	127.84	6	2.30E–37
29609721	*S. avermitilis*	247	D	213, 149	64	130.48	6	7.80E–30
297161230	*S. bingchenggensis*	237	D	203, 139	64	128.67	6	6.10E–33
337765279	*S. cattleya*	259	D	226, 162	64	130.11	6	9.10E–33
**4539208**	***S. coelicolor*** **(** ***SCO2139*** **)**	**256**	**D**	**221, 157**	**64**	**128.18**	**6**	**2.50E−31**
178467673	*S. griseus* subsp*. griseus*	249	D	217, 153	64	131.13	6	5.30E−34
374100011	*S. hygroscopicus jinggangensis*	225	D	190, 126	64	127.83	6	4.10E−30
197720707	*S. pristinaespiralis*	255	D	222, 158	64	128.94	6	3.30E–31
260650624	*S. scabiei*	251	D	209, 145	64	128.36	6	8.30E–30
297199120	*S. sviceus*	256	D	221, 157	64	130.03	6	8.10E−30
178466512	*S. griseus* subsp*. griseus*	249	E	181, 129	52	122.50	6	5.20E−25
197722368	*S. pristinaespiralis*	196	E	176, 123	53	125.71	6	6.40E−23
290958287	*S. scabiei*	223	E	198, 145	53	127.68	6	2.30E−20
197711057	*S. sviceus*	197	E	175, 122	53	127.02	6	1.20E−21

Bold characters are used to identify *S. coelicolor* genes.

^a^Amino acids.

^b^Active site.

^c^Transmembrane helix.

**Table 3:** Divergence across rhomboid families

Rhomboid type	A Strepto	B Strepto	C Strepto	D Strepto	E Strepto	E Myco	AB Myco
A Strepto	–						
B Strepto	0.29474	–					
C Strepto	0.35789	0.35789	–				
D Strepto	0.40001	0.38947	0.16842	–			
E Strepto	0.43158	0.38947	0.40000	0.42105	–		
E Myco (Rv1337)	0.46316	0.53684	0.48421	0.50526	0.29474	–	
AB Myco (Rv0110)	0.23158	0.29500	0.33684	0.36842	0.35789	0.51579	–

This table was built using consensus sequences representative of each family.

**Figure 3:**
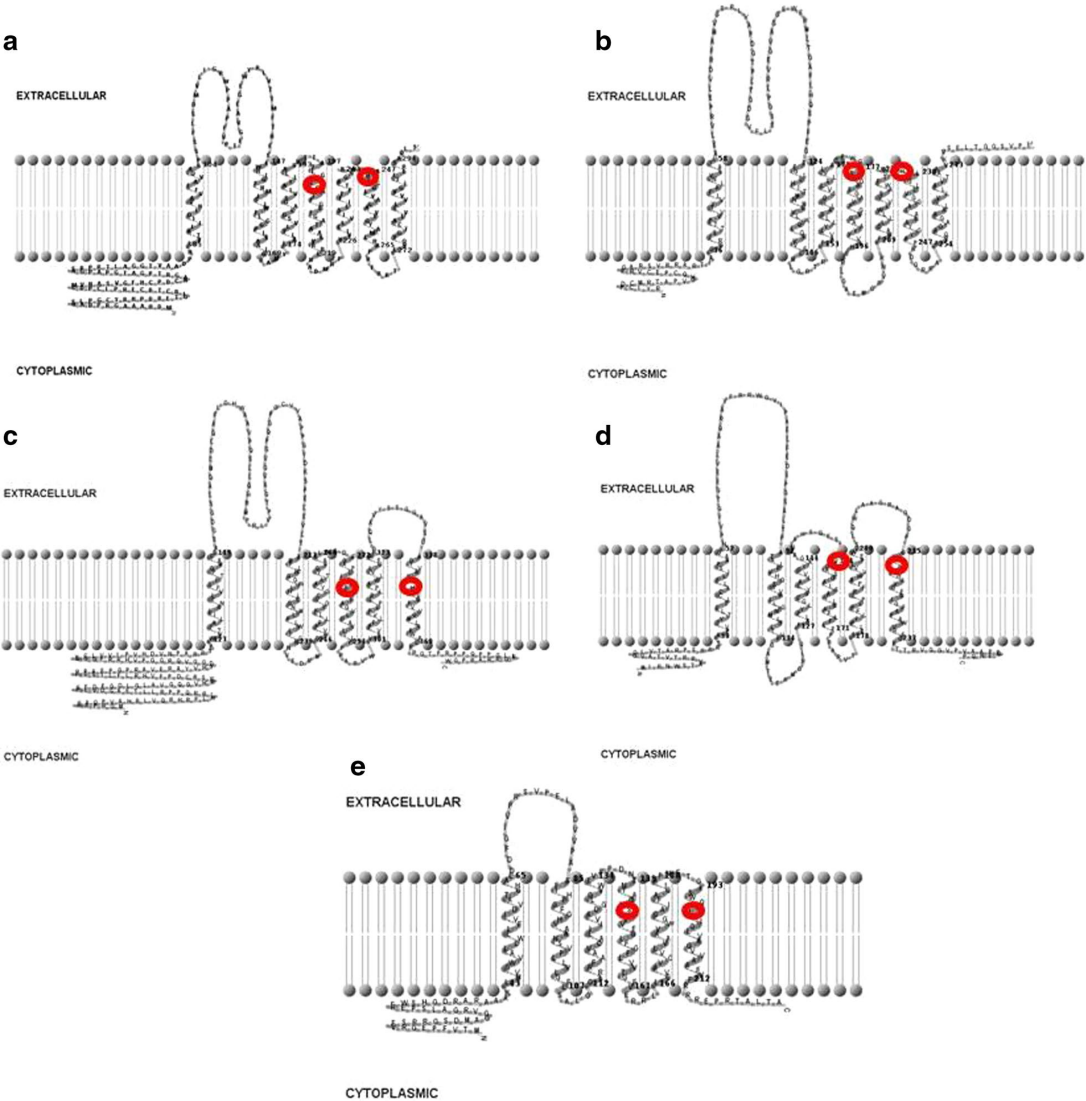
Transmembrane structure of Rhomboids using TMRPres2D [45]. Rhomboids** a**,** b**,** c** and** d** are from *S. coelicolor*, and ** e** is from *S. sviceus* active sites are highlighted with a *red circle.*

The topological analysis of the *Streptomyces* rhomboid genes shows that families C, D and E have the most prevalent structure in prokaryotic rhomboids, 6 TM helices; interestingly families A and B have an additional TM helix similar to AarA from *P. stuartii* [7] and YqgP from *B. subtilis* [8] (Figure 3). It has been suggested that the bacterial rhomboids with the 6 + 1 TM helices could either be a bacterial progenitor to the eukaryotic rhomboid proteases or they may represent an ancient family of rhomboid proteases present in the last universal common ancestor (LUCA) [45]. The fact that family A (6 + 1 TM topology) and family D (6 TM topology) are present in all genomes analyzed suggests that each family could have different and potentially critical biological roles in *Streptomyces*.

We have also found that families A and B have zinc fingers as extra membranous domains, but this motif is less conserved in families C, D and E. It is suggested that these soluble accessory domains may be involved in the oligomerization status of the rhomboid proteins [46]. These findings again support the idea that different families could have distinct functions in *Streptomyces* biology.

**Figure 4:**
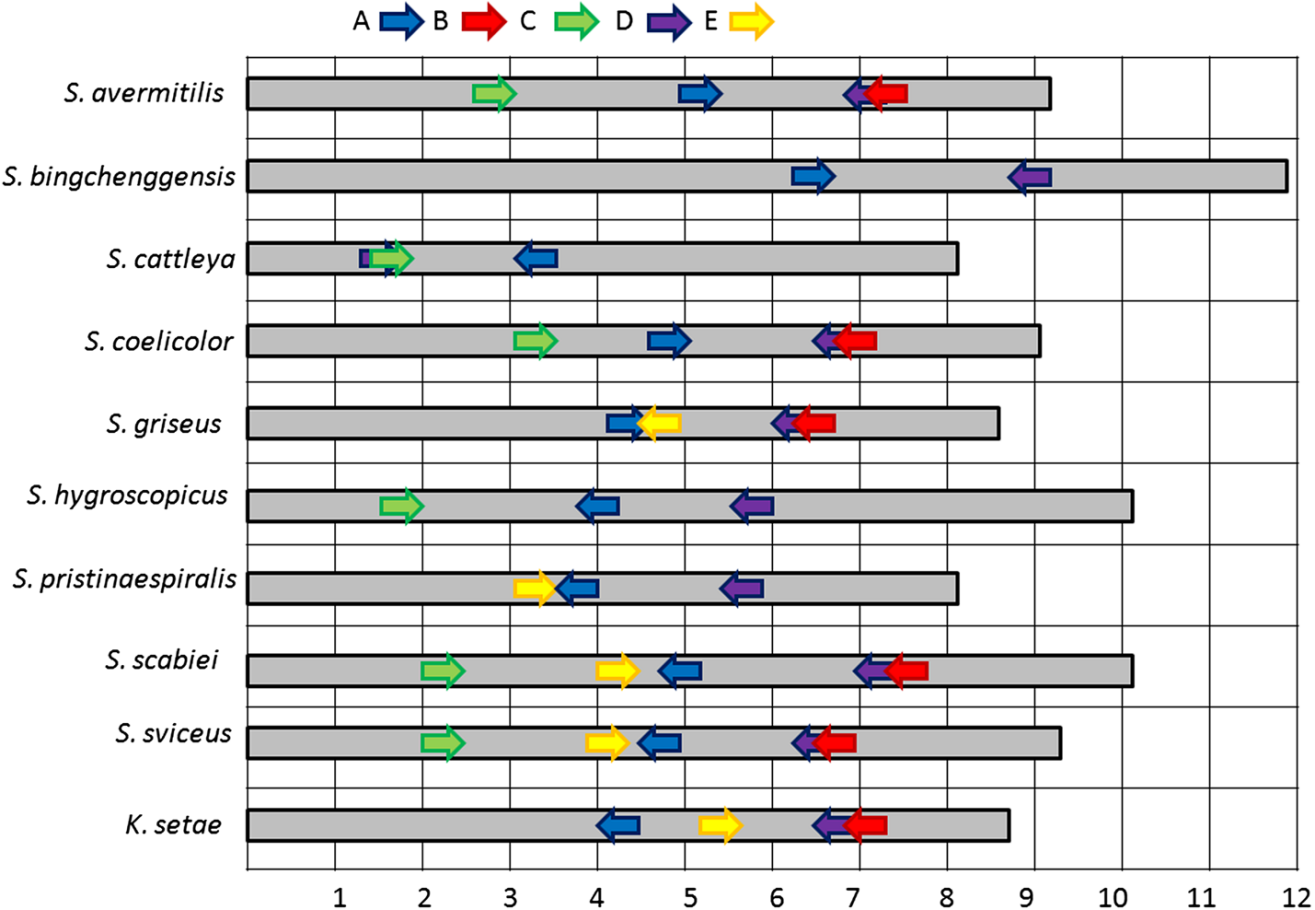
Location and orientation of rhomboids from selected species. Each family is *color* coded as in Figure 1.

*Family A* Rhomboids belonging to family A are orthologous to the Rv0110 rhomboid protease 1 in *Mycobacteria* [11]; they are of similar length and structure. Family A rhomboids have seven TM helices, with a long run of extracellular amino acids between helices 1 and 2. At the N-terminus, there is a long string of cytoplasmic amino acids that in many cases have a zinc-finger domain. Each protein contains the same catalytic residues, a serine and a histidine, 46 amino acids apart in TM 4 and 6 (Figure 3). Phylogenetic analysis indicates vertical transfer of rhomboid A gene (Figure 1) during the diversification of Actinomycetes (shown in blue), since they are found in the *Franki*a lineage, *Kitasatospora setae*, *Acidothermus cellulolyticus*, and in the nine *Streptomycetes* sequences analyzed. Rhomboid A genes are 27% divergent (Table 4), are found next to the gene encoding peptidyl-prolyl cis–trans isomerase, and the surrounding neighborhood is conserved across species (Figure 2). Usually they are located towards the center of the genome, but their orientation is not the same across all species (Figure 4). Genomic rearrangements could have contributed to the shuffling of its genomic location in some species.

**Table 4:** Average divergence of nucleotide sequences within rhomboid families

Rhomboid	Distance	SE
Family A	0.2721	0.0083
Family B	0.2732	0.0089
Family C	0.2065	0.0086
Family D	0.2348	0.0090
Family E	0.268	0.013

*Family B* Family B rhomboids are similar to family A in that they have 7 TMs (Figure 3), approximately 46 amino acids separating the active sites and are also 27% divergent (Table 4). B rhomboids are only found in *Streptomyces* and their sister species *Kitasatospora seta* and *Streptosporangia roseum* (Figure 1 shown in red). This is likely due to a gene duplication event before the diversification of the *Streptomyces* genus, but after the separation of other *Actinomycetes*, such as members of the genus *Frankia* and *Mycobacteria*. Although rhomboids A and B are paralagous, their 29% divergence (Table 3) is indicative of the antiquity of the duplication event, and may suggest that they have different roles. The location and orientation of rhomboid B (Figure 4) is highly conserved across *Streptomycetes*; it is located at the end of the genome and in close proximity to the rhomboid D gene. Rhomboid B genes have been lost in several species including, *S. bingchenggensis*, *S hygroscopicus* and *S. pristinaespiralis*. The phylogenetic history among species of *Streptomyces* is not well understood, and thus it is unknown if the loss of this gene was due to a single or multiple events.

**Figure 5:**
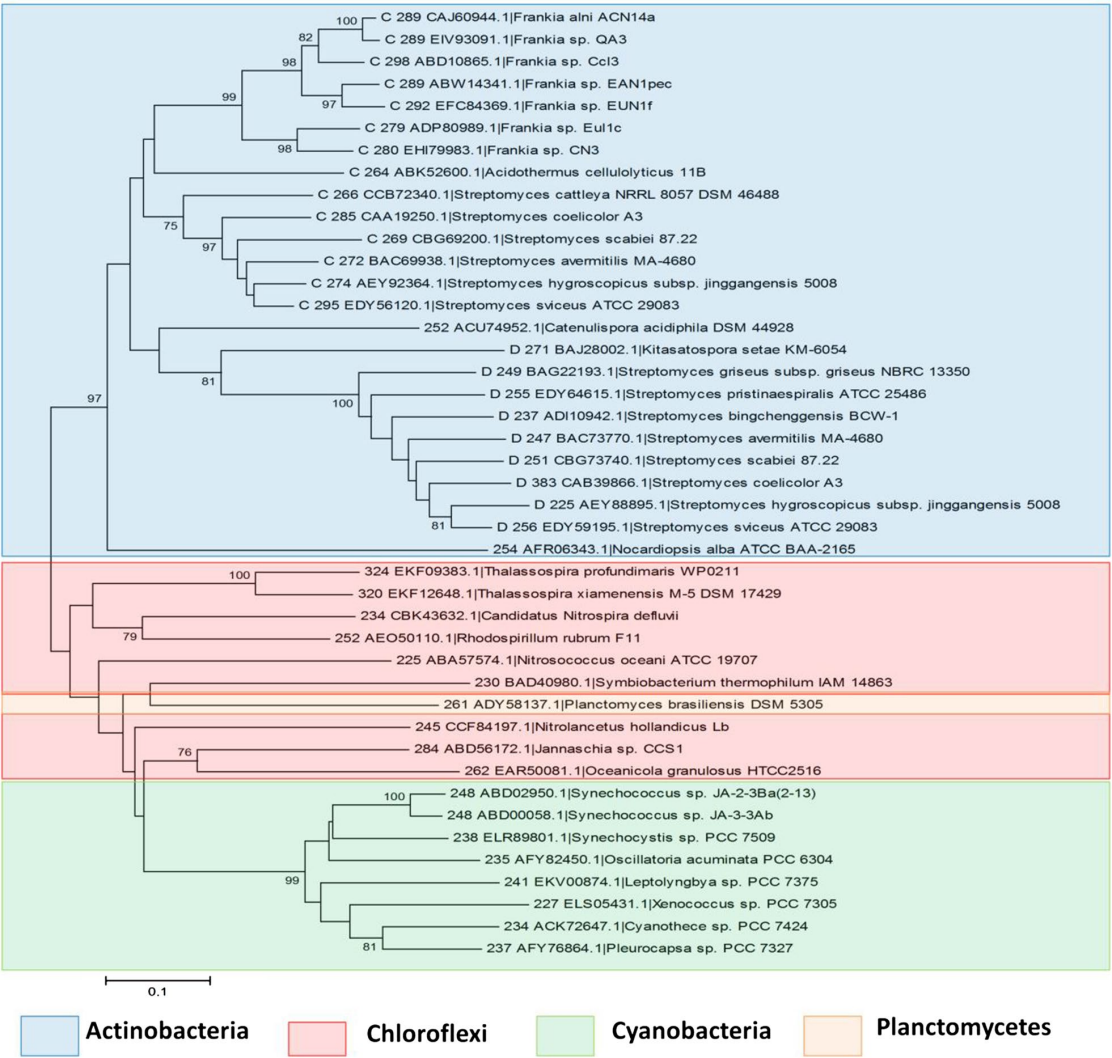
NJ Tree of rhomboids C and D using Mega 5.0. (Bootstrap values <70 have been removed). Rhomboids C and D are more similar to rhomboid genes in *Chlorofexi*, *Cyanobacteria* and *Planctomycetes* than they are to other rhomboids within the *Streptomycetes.*

*Families C and D* We hypothesize that the C and D rhomboid families are paralogous xenologs; these families are most likely due to a horizontal transfer event to the ancestor of the *Streptomyces*, *Kitasatospora*, *Frankia* and *Acidothermus* genera (Figure 1 shown in green and purple). Rhomboids C and D are, in addition, phylogenetically discontinuous, this is supported by their absence from the *Mycobacterium* lineage and other Actinobacteria. Since *Streptomycetes* are typically promiscuous, horizontal transfer of this lineage of rhomboids is possible; although it is also possible that they have been deleted from other Actinomycetes lineages several times. Families C and D are 80 and 77% similar within their families, respectively (Table 4). Further analysis of C and D rhomboids reveals a non-Actinomycetes ancestor (Figure 5) suggesting a different evolutionary history for these genes.

Rhomboids belonging to family C are found in six of the nine analyzed *Streptomycetes* (Table 2) and display a unique 6 TM motif with 66 amino acids separating the active sites (Figure 3). All C rhomboids are found towards the beginning of the genome when present, and in the same orientation (Figure 4).

D rhomboids also have 6 TMs, but consistently have only 64 amino acids separating their active sites (Figure 3). D rhomboids are found in all of the species analyzed, and therefore are likely to be functionally important. They are found at the end of the genome, and in reverse orientation. *S. cattleya* appears to be unique since its D rhomboid gene is found at the opposite end of the genome, in close proximity to the C gene, and in the forward orientation (Figure 4). This could be due to genomic rearrangement. As rhomboids C and D genes are the result of a duplication event in the *Streptomyces* ancestor, it is expected that all genomes that contain rhomboid D would also possess rhomboid C. However, rhomboid C is not present in three of the nine species analyzed. As discussed for the rhomboid B family, it is unknown if the loss of this gene was due to a single or multiple events.

*Family E* E rhomboids are orthologous to the Rv1337 rhomboid protease 2 in *Mycobacteria.* This family is found only in four of the nine genomes analyzed, with 27% divergence in their nucleotide sequence (Table 4). They are located in the middle of the linear genome, although the orientation is not conserved (Figure 4). They are also not found in the same operon or gene neighborhood as *Mycobacteria* homologs (Figure 2). Phylogenetic analysis indicates vertical transfer (Figure 1) during the diversification of *Actinomycetes*, as rhomboid E genes (shown in yellow) are found in *Actinomycetes*. Like other rhomboid genes, E rhomboids have been lost in several species, including *S. coelicolor* (Figure 2 shows the location where rhomboid E should be in the *S. coelicolor* genome). Their sequence analysis shows a structure of 6 TM helices with catalytic residues in TMs 4 and 6 separated by about 52 amino acids (Table 2).

### B. Transcriptional analysis of putative *S. coelicolor* rhomboid genes

**Figure 6:**
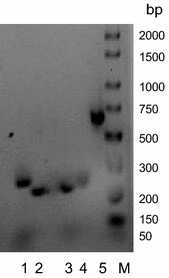
Amplification of internal fragments of *S. coelicolor* rhomboid genes. Genomic DNA was used as template and primers are listed in Table 1. *Lanes* *1* SCO2013; *2* SCO2139; *3* SCO6038; *4* SCO3855; *5* positive control using act primers; *M* exACTGene low range DNA ladder.

The existence of multiple rhomboid genes in *Streptomycetes* raises the question whether all, none, or some of them are transcribed. Phylogenetic analysis identified four candidate rhomboid genes (A, B, C and D) in *S. coelicolor*. Primers (Table 1) were designed to amplify internal fragments for these genes. Fragments of the predicted sizes were obtained using these primers and *S. coelicolor* genomic DNA as a template (Figure 6).

**Figure 7:**
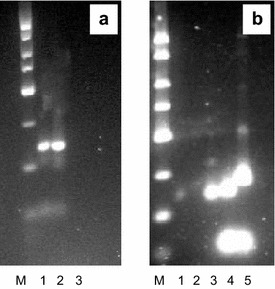
RT-PCR amplification of internal fragments of *S. coelicolor* rhomboid families. **a** *Lanes* *M* exACTGene low range DNA ladder; *1* SCO3855; *2* SCO3855; *3* negative control, *S. coelicolor* mRNA, not transcribed. **b** *Lanes* *M* exACTGene low range DNA ladder; *1* negative control *S. coelicolor* mRNA, not transcribed; *2* negative control (water); *3* SCO2139; *4* SCO6038; *5* SCO2013.

To determine if these genes are transcribed, we isolated mRNA from a *S. coelicolor* liquid culture, and used reverse transcriptase (RT) to make cDNA. Detection of transcribed rhomboid genes was done by PCR using the primers described above (Table 1). The expected fragment sizes were obtained (Figure 7). The purified PCR products were cloned into pUC19, and the correct inserts were verified by sequencing. Therefore, reverse transcription analysis confirms that the four rhomboid genes (SCO3855, SCO2139, SCO6038, SCO2013) are transcribed in *S. coelicolor*.

## Conclusions

In summary, our analysis demonstrates the existence of five distinct families of rhomboid genes in *Streptomycetes*. Families A and D are present in all *Streptomyces* genomes analyzed, family D displays the typical prokaryotic topology with 6 TMs while family A has 6 + 1 TMs. These findings suggest that both families A and D are essential and may play different biological roles in *Streptomyces*. The evolutionary dynamic of the *Streptomyces* rhomboids is very complex, and we will expand our analysis to other *Streptomyces* strains and the Actinobacteria taxa to obtain a more comprehensive understanding of it. We also show that the four rhomboid genes present in *S. coelicolor* are transcribed. We are currently studying the expression of these genes, specifically by constructing knock out (KO) mutants for each of the putative rhomboid genes from *S. coelicolor* and comparing the KOs to the wild type strain. This will provide insight into the involvement of rhomboids in *Streptomyces* physiology.
